# Leveraging digital twins for improved orthopaedic evaluation and treatment

**DOI:** 10.1002/jeo2.70084

**Published:** 2024-11-10

**Authors:** Michael C. Dean, Jacob F. Oeding, Pedro Diniz, Romain Seil, Kristian Samuelsson, ESSKA Artificial Intelligence Working Group

**Affiliations:** ^1^ School of Medicine Mayo Clinic Alix School of Medicine Rochester Minnesota USA; ^2^ Department of Orthopaedics, Institute of Clinical Sciences, The Sahlgrenska Academy University of Gothenburg Gothenburg Sweden; ^3^ Department of Orthopaedic Surgery Centre Hospitalier de Luxembourg—Clinique d'Eich Luxembourg Luxembourg

**Keywords:** artificial intelligence, augmented reality, deep learning, digital twin, orthopaedics

## Abstract

**Purpose:**

The purpose of this article is to explore the potential of digital twin technologies in orthopaedics and to evaluate how their integration with artificial intelligence (AI) and deep learning (DL) can improve orthopaedic evaluation and treatment. This review addresses key applications of digital twins, including surgical planning, patient‐specific outcome prediction, augmented reality‐assisted surgery and simulation‐based surgical training.

**Methods:**

Existing studies on digital twins in various domains, including engineering, biomedical and orthopaedics are reviewed. We also reviewed advancements in AI and DL relevant to digital twins. We focused on identifying key benefits, challenges and future directions for the implementation of digital twins in orthopaedic practice.

**Results:**

The review highlights that digital twins offer significant potential to revolutionise orthopaedic care by enabling precise surgical planning, real‐time outcome prediction and enhanced training. Digital twins can model patient‐specific anatomy using advanced imaging techniques and dynamically update with real‐time data, providing valuable insights during surgery and postoperative care. However, challenges such as the need for large‐scale data sets, technological limitations and integration issues must be addressed to fully realise these benefits.

**Conclusion:**

Digital twins represent a promising frontier in orthopaedic research and practice, with the potential to improve patient outcomes and enhance surgical precision. To enable widespread adoption, future research must focus on overcoming current challenges and further refining the integration of digital twins with AI and DL technologies.

**Level of Evidence:**

Level V.

**Abbreviations**
AI: artificial intelligence
AR: augmented reality
CT: computed tomography
DL: deep learning
MRI: magnetic resonance imaging
PET: positron emission tomography
RPM: remote patient monitoring
VR: virtual reality

## INTRODUCTION

Recent developments in artificial intelligence (AI) and deep learning (DL) have enabled several advancements within orthopaedics, including outcome forecasting with predictive modelling [14, 15, 23], automated annotation of imaging studies [19, 24, 26], compilation of synthetic data using generative AI [16] and establishment of rich registries via efficient data mining [25, 30]. Another promising application of AI is the use and analysis of digital twins. Although digital twin technologies have been widely deployed in engineering, industrial and biomedical domains, their implementation in orthopaedics remains relatively limited.

Digital twins are virtual models of physical entities that are continuously updated with real‐time data. They integrate various technologies—including real‐time data collection, simulation and predictive analytics—to create accurate and dynamic representations of physical objects, systems or processes. For example, the Apollo 13 mission is famously cited as the first application of digital twin technologies [10]. Following an in‐flight explosion damaging the spacecraft's main engine, NASA mission controllers used data from the compromised spacecraft to modify and dynamically evolve earth‐bound simulators, ultimately enabling the astronauts to return home safely. This example highlights an important distinction between digital twins and traditional model simulations: rather than existing as static models, digital twins are iteratively updated with real‐time data from their physical counterparts, enabling more nuanced predictive insights.

Digital twins hold significant potential in addressing orthopaedic research questions and improving patient care and surgical outcomes, particularly when combined with novel AI developments discussed previously. In this review, we discuss four promising applications of digital twins in orthopaedics: surgical planning and simulation, patient‐specific prediction of postoperative outcomes, augmented reality (AR)‐assisted surgery and simulation‐based surgical training. Finally, we conclude with current challenges hindering the implementation of digital twins in orthopaedics, as well as future directions that may enable widespread deployment of these technologies.

## SURGICAL PLANNING AND SIMULATION

Leveraging advanced imaging studies such as magnetic resonance imaging (MRI) and computed tomography (CT) scans, digital twins can be constructed to precisely model a patient's specific anatomy, which can then be used to plan particularly complex procedures. For example, a proof‐of‐concept study employed digital twins to model and analyse the atria of patients with persistent atrial fibrillation and fibrosis. Specifically, Boyle and colleagues synthesised heart structure, fibrosis and inflammation identified through MRI and positron emission tomography scans and electrophysiology studies to create personalised virtual models to identify catheter ablation targets [3]. In the context of orthopaedics, multidisciplinary teams could similarly integrate MRI and CT scans to create digital twins that capture a patient's specific bony morphology and soft tissue pathology to plan complex multiligamentous knee injuries, for example. Indeed, in a systematic review by Bjelland et al., the authors propose a conceptual digital twin system that integrates patient‐specific MRI data, real‐time intraoperative sensor information and advanced biomechanical modelling to simulate arthroscopic knee surgery with high accuracy [2]. These digital twins hold potential for orthopaedic surgeons at various levels of expertise. For junior residents, they offer a safe, realistic platform to practice technically challenging procedures through repeated simulations of patient‐specific cases. For more experienced surgeons, they serve as a valuable tool for preoperative planning and rehearsal of particularly complex or rare cases. Furthermore, instead of merely allowing the execution of a single preplanned procedure, these digital twin models can be refined and repeated with different surgical approaches, graft choices and fixation techniques, thus revealing the safest and most effective surgical plan for the patient. Notably, this surgical planning approach could be further enhanced by recent DL‐based pipelines capable of generating accurate 3D bony reconstructions using MRI alone, eliminating the need for multiple imaging studies [13, 29].

## PATIENT‐SPECIFIC PREDICTION OF POSTOPERATIVE OUTCOMES

The creation of preoperative digital twins incorporating patient demographics, specific anatomy and surgical intervention represents the first step in optimising postoperative outcomes; however, these virtual models would fail to reach their full potential without continuous integration of postoperative data. Specifically, each preoperative digital twin could be dynamically updated with patient‐specific, real‐time data from the recovery process, including patient‐reported outcome measures, rehabilitation adherence and sensor data from wearable devices. Such remote patient monitoring (RPM) programmes have received growing attention within orthopaedics, particularly following total hip and knee arthroplasty [18, 21, 22, 28]. Of note, existing literature on RPM programmes in orthopaedics have primarily relied on patient surveys and simple range‐of‐motion measurements. However, RPM protocols could be significantly enhanced by adopting digital twin technologies widely applied in other industries, such as aerospace and automotive engineering.

A notable example is the use of digital twins in the aerospace industry for predictive maintenance. For instance, aircraft manufacturers such as Boeing and Rolls‐Royce have implemented digital twins to monitor the performance of engines and other components in real time, using sensor data and machine‐learning algorithms to predict wear and tear or potential malfunctions before they occur [1, 12]. By continuously updating the digital model with real‐time data from the physical system, they can foresee maintenance needs and optimise operational performance, preventing costly failures.

This predictive maintenance approach could be translated into orthopaedic surgery and postoperative care. Just as aerospace engineers use digital twins to prevent in‐flight engine failures, orthopaedic surgeons could leverage digital twins to monitor joint implants or surgical outcomes in real time. Recently, hospitals such as the Hospital for Special Surgery and Mayo Clinic have begun to implement smart orthopaedic implants for knee replacements [9, 11]. These implants use embedded sensors to capture patient‐specific data—such as movement patterns, load distribution, range of motion and other gait‐related metrics—which are transmitted to the care team in real time. This real‐time data allows orthopaedic surgeons to customise postoperative care and adjust rehabilitation protocols based on objective feedback from the smart implant, potentially enhancing recovery outcomes. However, while these pilot studies demonstrate the safety and feasibility of this technology, further research is needed to evaluate RPM's potential to predict outcomes and guide targeted intervention that prevent complications before they arise.

## AR ASSISTED SURGERY

Digital twins can also be utilised in AR‐assisted surgery by providing real‐time, precise visualisations of a patient's anatomy during procedures [2, 7]. Leveraging technologies discussed previously, MRI with or without CT can be used to generate patient‐specific anatomical models, including bony morphology, soft tissues and notable neurovascular structures [13, 29]. This digital twin is then superimposed onto the surgeon's view of the operative field using an AR headset, enabling ultraprecise implant placement and safe navigation around critical structures. For example, in a recent cadaveric study evaluating the use of AR navigation in total shoulder arthroplasty, Sanchez‐Sotelo et al. demonstrated high accuracy and precision of placement of the glenoid axis pin employing CT‐generated 3D reconstructions and an AR headset [27]. Another study implementing AR guidance on real patients similarly found improved accuracy and precision of glenoid positioning compared to standard instrumentation (i.e., placement of guide pin using anatomic landmarks), highlighting clinical feasibility of AR technology [17]. Although not necessary in these two applications, the utility of AR assistance could be further augmented via continuous updating of the virtual model, for example, during acetabular reaming in hip arthroplasty. This dynamic feedback loop would allow surgeons to adjust instrument placement in real time, improving outcomes and minimising complications.

## SIMULATION‐BASED SURGICAL TRAINING

The landscape of orthopaedic surgery resident education is constantly evolving, with growing use of surgical simulation and procedural skills laboratories to complement supervised surgery in the operating room. Several studies have demonstrated the effectiveness of virtual reality (VR) simulators to improve residents' performance of knee arthroscopy, total shoulder arthroplasty and shoulder arthroscopy [5, 6, 8, 20]. Studies comparing skill acquisition with cadaver laboratories versus VR simulators remain mixed, with Camp and colleagues revealing faster improvement with cadaveric specimens and Crockatt et al. finding no significant difference [5, 8]. It seems plausible that one advantage of cadaver training is representation of anatomical variations and unique pathologies, as opposed to a standardised model. However, implementation of digital twins may help bridge this gap. Specifically, the standardised models currently employed by VR simulators could be updated with real‐life, patient‐specific MRI and/or CT scans, more accurately capturing anatomical variations and pathological conditions. Moreover, use of digital twin technologies would provide the additional benefit of enabling residents to complete the same case in both the virtual and physical world. Although future research is needed to rigorously test this hypothesis, we speculate that performing the case in both environments would both improve execution of the real‐life surgery and enhance transferability of skills from the training setting to live surgery.

## CONCLUSIONS

The implementation of digital twins in orthopaedics offers significant potential for enhancing surgical planning, predicting postoperative outcomes, optimising AR‐assisted surgery and advancing resident education through simulation‐based training. Despite these promising applications, several challenges must be addressed before they are fully realised. Perhaps most importantly, each advancement relies on high‐quality, large‐scale data sets to train and test the underlying AI models. Continuing the multiligamentous knee injury example described previously, simulation of different surgical approaches, graft choices and fixation techniques on a given virtual twin would require an accurate risk calculator developed using rich clinical data, similar to a recently published risk calculator predicting dislocation risk following hip arthroplasty [15]. In fact, supporting efforts to curate comprehensive data sets—including pre‐ and postoperative imaging and patient outcomes—may represent the single most impactful opportunity for orthopaedic surgeons to promote further development of digital twin technologies. Moreover, continued optimisation of AR‐assisted surgery and VR‐based training depends upon technological advancements, such as improved accuracy and reliability of AR systems in diverse settings and incorporation of realistic haptic feedback to VR simulators [4, 27]. As digital twin technologies continue to progress, their widespread adoption holds the potential to both enable innovative orthoepic research and promote the delivery of safer, individualised treatments.

## AUTHOR CONTRIBUTIONS

**Michael C. Dean**: Study design; data acquisition; data analysis; data interpretation; manuscript drafting; critical revision. **Jacob F. Oeding**: Study design; data acquisition; data analysis; data interpretation; manuscript drafting; critical revision. **Pedro Diniz**: Study design; data acquisition; data analysis; data interpretation; manuscript drafting; critical revision. **Romain Seil**: Study design; data acquisition; data analysis; data interpretation; manuscript drafting; critical revision. **Kristian Samuelsson**: Study design; data acquisition; data analysis; data interpretation; manuscript drafting; critical revision.

## CONFLICT OF INTEREST STATEMENT

The authors declare no conflict of interest.

## ETHICS STATEMENT

The authors have nothing to report.
